# Towards system genetics analysis of head and neck squamous cell carcinoma using the mouse model, cellular platform, and clinical human data

**DOI:** 10.1002/ame2.12367

**Published:** 2023-12-21

**Authors:** Osayd Zohud, Iqbal M. Lone, Aysar Nashef, Fuad A. Iraqi

**Affiliations:** ^1^ Department of Clinical Microbiology and Immunology, Sackler Faculty of Medicine Tel‐Aviv University Tel Aviv Israel; ^2^ Department of Oral and Maxillofacial Surgery Baruch Padeh Medical Center Poriya Israel; ^3^ Azrieli Faculty of Medicine Bar‐Ilan University Ramat Gan Israel

**Keywords:** animal models, Collaborative Cross mice, genomics, head and neck squamous cell cancinoma, host genetic susceptibility

## Abstract

Head and neck squamous cell cancer (HNSCC) is a leading global malignancy. Every year, More than 830 000 people are diagnosed with HNSCC globally, with more than 430 000 fatalities. HNSCC is a deadly diverse malignancy with many tumor locations and biological characteristics. It originates from the squamous epithelium of the oral cavity, oropharynx, nasopharynx, larynx, and hypopharynx. The most frequently impacted regions are the tongue and larynx. Previous investigations have demonstrated the critical role of host genetic susceptibility in the progression of HNSCC. Despite the advances in our knowledge, the improved survival rate of HNSCC patients over the last 40 years has been limited. Failure to identify the molecular origins of development of HNSCC and the genetic basis of the disease and its biological heterogeneity impedes the development of new therapeutic methods. These results indicate a need to identify more genetic factors underlying this complex disease, which can be better used in early detection and prevention strategies. The lack of reliable animal models to investigate the underlying molecular processes is one of the most significant barriers to understanding HNSCC tumors. In this report, we explore and discuss potential research prospects utilizing the Collaborative Cross mouse model and crossing it to mice carrying single or double knockout genes (e.g. *Smad*4 and P53 genes) to identify genetic factors affecting the development of this complex disease using genome‐wide association studies, epigenetics, microRNA, long noncoding RNA, lncRNA, histone modifications, methylation, phosphorylation, and proteomics.

## Graphical abstract

Head and neck squamous cell cancer (HNSCC) is the sixth most frequent cancer worldwide. Annually, there are more than 830 000 individuals diagnosed with HNSCC globally, and more than 430 000 patients die of this disease. HNSCC is a fatal heterogeneous disease with highly variable tumor sites and biological behaviors. It arises from the squamous epithelium of the oral cavity, oropharynx, nasopharynx, larynx, and hypopharynx, their hypothetical locations. The tongue and larynx are the most often affected areas. This cancer can cause irregular patches or ulcers in the mouth and throat, along with unusual bleeding or pain, depending on the location. Additionally, it was shown that it might lead to persistent sinus congestion, sore earache, and pain or difficulty in swallowing, with other complications including a hoarse voice, breathing difficulty, or enlarged lymph nodes. The classical risk factors for HNSCC are smoking and excessive alcohol use. Viral infections, including human papillomaviruses (HPV) and Epstein–Barr virus (EBV) cause a substantial and rising proportion of these tumors. Previous studies have shown the crucial role of host genetic susceptibility in HNSCC development. Despite the advances in our knowledge of epidemiology and pathogenesis, the improved survival rate of HNSCC patients over the past four decades has been limited. The lack of identifying the molecular carcinogenesis of HNSCC development and the genetic basis of the disease and biological heterogeneity impedes the development of new therapeutic methods. These results indicate a need to enhance the identification of more genetic factors underlying this complex disease, which can be better sued in early detection and prevention strategies. The lack of reliable animal models to explore the underlying molecular mechanisms is one of the most severe impediments to better understanding the HNSCC tumors. In this report, we address HNSCC and discuss future research opportunities using the Collaborative Cross (CC) mouse model and crossing it to mice carrying single or double knockout genes (e.g. *Smad*4 and P53 genes) to comprehensively identify genetic factors affecting the development of this complex by studying genome‐wide association study (GWAS), epigenetics, small and microRNA, long noncoding RNA (lncRNA), lncRNA, Histone modifications, Acetylation, Methylation, phosphorylation, and Proteomics.

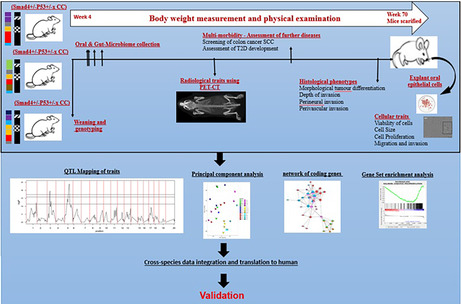

## 1 INTRODUCTION

**FIGURE 1:**
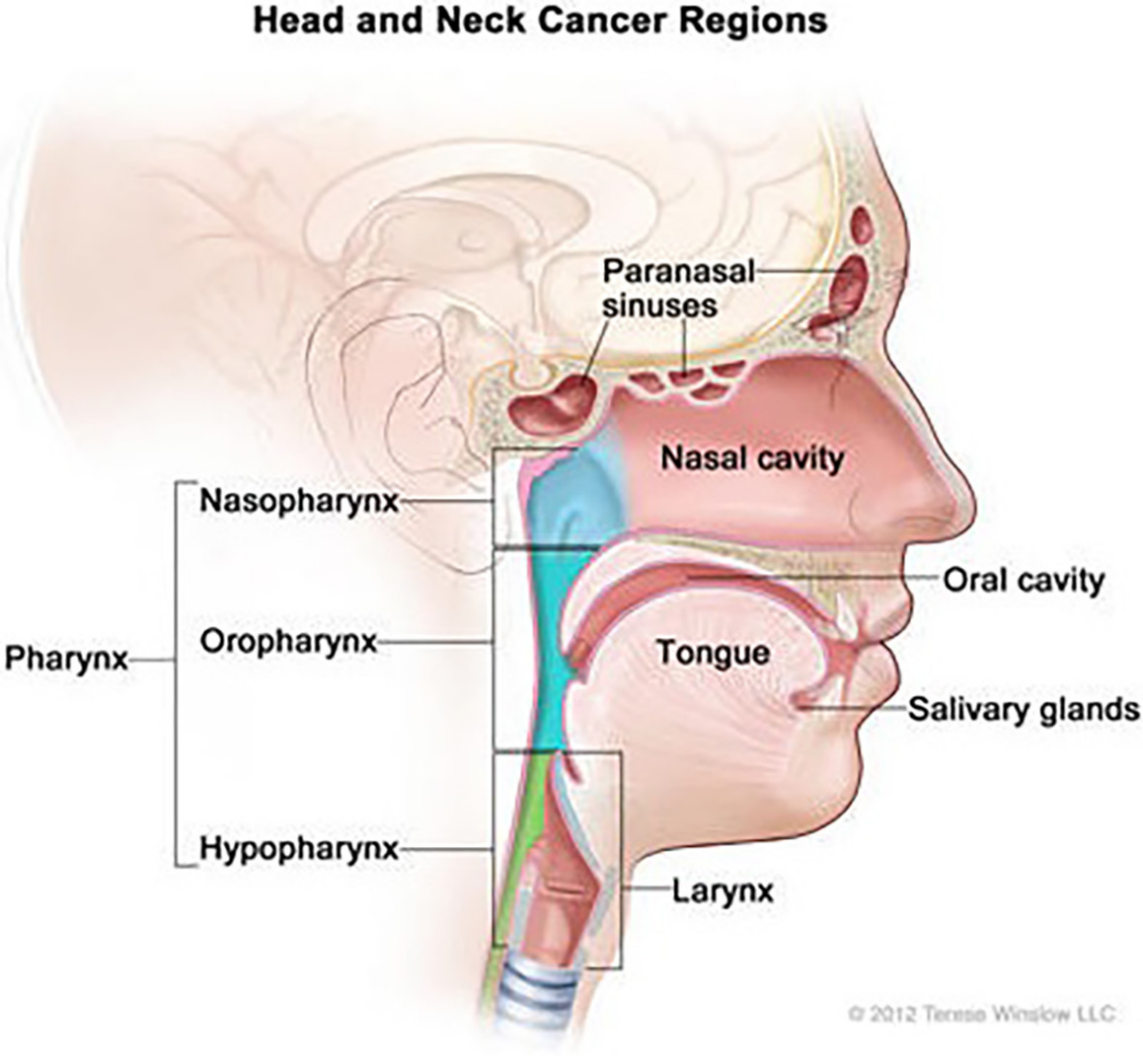
Schematic diagram showing the sites and tissues associated with human HNSCC.

Head and neck squamous cell carcinoma (HNSCC) is the sixth most common cancer in the world, and a primary source of illness and death in developing nations.^1^ Annually, there are over 830 000 people affected by HNSCC globally, and over 430 000 individuals die of this malignancy.^2^ HNSCC is a lethal heterogeneous disorder with a wide range of tumor sites and biological characteristics. It arises from the squamous epithelium of the oral cavity, oropharynx, nasopharynx, larynx, and hypopharynx,^3^ as shown in Figure 1. The tongue and larynx are the most often affected areas. This malignancy can cause irregular patches or ulcers within the mouth and throat, accompanied by abnormal bleeding or discomfort, depending on the location. Additionally, it was shown that it might lead to persistent sinus congestion, painful earache, and pain or difficulty in swallowing, with other complications including a raspy voice, breathing difficulty, or lymph nodes that have become swollen.^4^

Tobacco usage and excessive alcohol use are traditional risk factors for HNSCC. Viral infections, including human papillomaviruses (HPV) and Epstein–Barr virus (EBV), cause a significant and increasing proportion of these malignancies.^5^, ^6^ However, only a tiny percentage of those who use cigarettes, consume alcohol or have HPV acquire the disease,^7^ thus implying an essential part of host genetic vulnerability in the development of HNSCC. For instance, genetic variations in the alcohol‐associated genes *ADH1B* and *ADH7* were linked to an increased likelihood of HNSCC malignancies.^8^ Several genetic changes have been observed in HNSCC patients, including deletion of genetic material on 9p (which codes for p16), 17p (which codes for p53), 18q (which codes for DCC, Smad4, and Smad2,^9^, ^10^, ^11^ as well as the type II TGF‐ receptor (TGFBR2)).^12^ The upregulated oncogenic pathways in HNSCC patients are Ras, EGFR/Stat3, and PI3K/PTEN/Akt pathways.^13^ Ras activation is involved in the onset of HNSCC but is inadequate for malignant transformation.^14^

Despite breakthroughs in epidemiology and pathogenesis, the improved survival rate of HNSCC patients over the last 40 years has been limited.^15^ Patients with HNSCC have a 5‐year survival rate of about 50%.^16^ Patients with recurrent illness, bone invasion, or metastases have a dismal prognosis.^17^ HNSCC survivors are projected to account for 3% of all cancer survivors, with a 15% 5‐year mortality rate and a 42% 10‐year mortality rate.^18^ In HPV‐related tumors, the virus seems to have a significant impact on HNSCC survivability, with HPV‐positive patients having a 90% 5‐year survival rate.^19^ The recommended treatment method is amputation, which is linked with significant morbidity.^20^ As a result, novel techniques and research tools are required to advance drug‐based therapy choices.

Today a lack of knowledge about the molecular origins of HNSCC, along with the genetic underpinnings of this condition and biological heterogeneity, impedes the advancement of novel therapeutic approaches. The current Food and Drug Agency (FDA) authorized targeted therapies, including cetuximab, pembrolizumab, and nivolumab, have shown poor efficacy.^21^ Therefore, a better and deeper understanding of molecular pathways and mechanisms underlying HNSCC development is needed to identify novel biomarkers and more efficient treatments, which will provide better early prediction and subsequently map the road to early treatment and prevention strategies. It must be noted that identification of people at elevated risk for HNSCC based on epidemiological and clinical studies led to the recognition of a genetic role in this cancer type. Importantly, identifying host genes underlying this disease would allow identification people with an elevated risk of HNSCC, which would enhance our understanding of the molecular mechanisms driving the condition and support the discovery of therapeutic targets for HNSCC patients.

To our knowledge, there is no currently available syngeneic tumor model that faithfully depicts the cellular and molecular alterations linked with the onset and advancement of HNSCC tumors. The lack of reliable animal models for investigating the fundamental molecular mechanisms of HNSCC represents one of the most significant obstacles to better understanding HNSCC tumors. At the time of writing this review, there has been no comprehensive review of HNSCC or the accessible data spread throughout the literature discussing genetic, epigenetic and proteomic aspects of this disease. Thus, the aim of this report is to pull together the most recent information and publications on HNSCC. We will address HNSCC subtype models and their appropriateness for investigating genetic, epigenetic, and proteomic elements of HNSCC, and demonstrate the potential of systems genetics analysis to improve our understanding of human HNSCC, including subsites susceptibility, utilizing the Collaborative Cross (CC) mouse model.^22^, ^23^, ^24^, ^25^

### 1.1 Oropharynx SCC

The oropharyngeal squamous cell carcinomas (OPSCC) arise in the mucosal layer of the upper aerodigestive tract epithelium. Even though OPSCC may be developed from a solitary cell type within a specific tissue, it displays significant diversity. This diversity may be explained by variances in causative factors and the molecular changes that propel the process of carcinogenesis.

The incidence of OPSCC has increased significantly during the last 20 years, mainly owing to the remarkable increase in human papillomavirus (HPV)‐associated OPSCC cases.^26^ Besides etiological differences, HPV‐positive and HPV‐negative patients are clinically and biologically distinct. Patients with HPV‐related tumors are often younger, possess a higher socioeconomic status, and have lower primary and higher nodal stages of the illness than those with HPV‐negative tumors.^27^ HPV(+) status has a better prognosis; regardless of treatment approach, the 5‐year survival rates with HPV‐associated tumors are around 75%–80%, but only around 45%–50% for those with HPV‐negative tumors.^27^, ^28^ HPV‐associated oropharyngeal SCC occurs in <1% of oral HPV infections. There is no one common mutation in HPV‐positive oropharynx cancers; instead, numerous distinct mutations have resulted in the same malignancy. The single established phase in the process of carcinogenesis involves the binding of HPV oncoproteins E6 and E7 to tumor‐suppressor proteins p53 and pRb, leading to their inhibition. This results in uncontrolled cellular replication and disruption of the cell cycle.^29^ The HPV oncogene roles and the biology of SCCs have been extensively examined in both laboratory models and humans. However, the factors linked to the host tissue and immune response that determine an individual's vulnerability to infection and/or cancer development remain unknown.

The studies defining the breadth of genetic mutations in malignancies have improved our understanding of the changes involved in HNSCC tumorigenesis.^30^ A considerable amount of intertumoral heterogeneity demonstrates the biological complexity of these malignancies. Regardless of smoking status, HPV‐negative cancers have a high incidence of genetic mutations, whereas HPV‐positive tumors have considerably fewer mutated genes and genetic variations per tumor.^31^ The Cancer Genome Atlas (TCGA) consortium's study of the genetic makeup of 279 HNSCC cancers describes the molecular variations among HPV‐positive and ‐negative cases. HPV‐positive patients often exhibit activating mutations in the helical domain of PIK3CA, while HPV‐negative cases frequently display mutations in TP53 and CDKN2A.^32^ These discoveries imply that the lack of an oncogenic impact by HPV oncoproteins in tumors unrelated to HPV requires a buildup of several abnormalities to enable cancer development.^31^

### 1.2 Nasopharynx SCC

Nasopharyngeal carcinoma (NPC) is an exceptional squamous cell carcinoma involving epithelial cells, known for its marked invasive growth pattern and rapid onset of early distant metastasis. About 129 000 new instances of nasopharyngeal carcinoma were detected in 2018, accounting for 0.7% of all malignancies diagnosed in 2018. Epidemiological studies have shown that NPC affects both sexes, with a high sex ratio of 3:1 in favor of males,^33^ and affects individuals after 40.^34^ The various factors being suggested for its wide geographic distribution, including Epstein–Barr virus (EBV) infection, host genetics, and environmental factors such as tobacco use (both active and passive), intake of preserved foods, alcohol, and oral hygiene.^35^

The primary treatment of NPC is radiotherapy and complementary chemotherapy. However, frequent local recurrence and distant metastases remain the leading causes of treatment failure.^36^ Therefore, more research to investigate the molecular mechanisms of NPC and cancer invasion is needed. One of the significant factors that have impeded the advancement of NPC research is limited EBV‐positive models.^37^ Recently, several patient‐derived xenografts (PDX) with Epstein–Barr virus (EBV) positivity were effectively cultured within non‐obese diabetic‐severe combined immunodeficiency (NOD SCID) mice.^38^ However, these models offer restricted insights for immune‐oncology research because of the lack of a human immune response.^39^ To our knowledge, only one report describes the creation and analysis of a humanized mouse model for NPC‐PDX.^38^ As a result, there is an urgent need to develop and ultimately define a dependable and resilient human‐into‐mouse platform for nasopharyngeal cancer (NPC) to enable further research into NPC biology and screening and testing next‐generation combination immunotherapy.

The role of genetic susceptibility and environmental factors in the advancement of nasopharyngeal carcinoma (NPC) has received a lot of attention.^40^ NPC has a remarkable ethnic and geographical distribution.^41^ In addition to the high prevalence of NPCs in southern China, the presence of many occurrences of NPC in first‐degree relatives and familial aggregations of the illness have also been reported, suggesting that genetic predisposition is implicated in the disease's causation.^42^ Human genome‐wide association studies (GWAS) are the research method for identifying genetic variations that are statistically related to the risk of a disease or a particular attribute.^42^ The summary of GWAS findings related to HNSCC is presented in Table 1.

**TABLE 1:** Human genome association studies have identified genes associated with OSCC.

Trait(s)	Samplesize	Ancestry	Mapped genes	Variant and risk allele	*p*‐value	Chromosome: Position in bp	Reference
Oral cavity cancer	6034 cases/6585 controls	European, North America, South America	AC074091.2, GPN1	rs6547741‐G	4 × 10−8	2:27633057	[7]
			ADH1B	rs1229984‐G	1 × 10−9	4:99318162	
			CLPTM1L	rs10462706‐C	6 × 10−10	5:1343679	
			CDKN2B‐AS1	rs8181047‐A	4 × 10−9	9:22064466	
			LAMC3	rs928674‐G	2 × 10−8	9:131076637	
			FAM227B	rs10851478‐G	4 × 10−7	15:49536822	
			NR2F2‐AS1	rs2398180‐T	3 × 10−7	15:96319940	
			HLA‐DQB1, MTCO3P1	rs3129780‐A	6 × 10−8	6:32679924	
			LTB, TNF	rs1800628‐A	7 × 10−8	6:31579073	
Pharynx cancer, oral cavity cancer			ADH1B	rs1229984‐G	2 × 10−15	4:99318162	
			AC106744.1	rs79767424‐C	4 × 10−10	5:19108581	
			TRIM5	rs1453414‐C	5 × 10−8	11:5807854	
			HLA‐DQB1	rs3828805‐C	3 × 10−13	6:32668343	
			LAMC3	rs7745247‐C	3 × 10−7	9:131087186	
Laryngeal SCC	2398 cases/2804 controls	East Asian	NCR3, UQCRHP1	rs2857595‐?	2 × 10−15	6:31600692	[51]
			FADS2, FADS1	rs174549‐?	1 × 10−20	11:61803910	
			ATF1P1, AL589947.1	rs9445023‐?	7 × 10−7	6:92812416	
			VCAN	rs310518‐?	7 × 10−8	5:83523300	
			RPL21P119, LINC02177	rs40129‐?	4 × 10−7	16:9344835	
			AC010183.2, LINC02459	rs10492336‐?	4 × 10−14	12:114147775	
Head and neck squamous cell carcinoma		East Asian	AL353133.2, AL157777.1	rs16879870‐?	4 × 10−6	6:86822028	[169]
			SCIMP, AC087500.1	rs2641256‐?	8 × 10−6	17:5223379	
			UQCRHP1, NCR3	rs2857595‐?	2 × 10−15	6:31600692	
			FADS1, FADS2	rs174549‐?	1 × 10−20	11:61803910	
			AL589947.1, ATF1P1	rs9445023‐?	7 × 10−7	6:92812416	
Oral cavity cancer	2171 cases/4493 controls	Non‐Hispanic whites	CLPTM1L	rs10462706‐C	8 × 10−11	5:1343679	[57]
			HLA‐DQB1	rs1049055‐T	3 × 10−9	6:32666610	
Oropharynx cancer			GALNT14	rs4318431‐T	3 × 10−9	2:30875199	
			MUC21	rs13211972‐A	1 × 10−10	6:30991224	
			HLA‐DQA1	rs34518860‐G	3 × 10−17	6:32626326	
Hypopharynx/larynx cancer			RTTN	rs142021700‐C	3 × 10−9	18:70034347	
			AKR1C1	rs77045180‐T	1 × 10−7	10:4981859	
HNSCC			HLA‐DQB1	rs3135001‐C	1 × 10−16	6:32702359	
			CCHCR1	rs1265081‐A	4 × 10−10	6:31143898	
			ZNRD1‐AS1	rs259919‐A	3 × 10−9	6:30057726	

Like many cancers, multiple genetic alterations were shown to be associated with NPC cases. Large‐scale case–control association studies and familial linkage investigations have revealed numerous susceptibility loci for NPC, thus substantiating the role of genetic factors in influencing the risk of NPC (data summarized in Table 2). Thus, characterizing the somatic mutations associated with NPC and their possible role in EBV regulation may give new insights into the pathophysiology of NPC. In addition, GWAS and whole exome sequencing (WES) analyses conducted on humans have pinpointed many NPC susceptibility loci with moderate risk on different chromosomes (data summarized in Table 2). However, the physiological mechanisms behind greater vulnerability to NPC and the link to EBV infection remain unknown.^43^

**TABLE 2:** Genome‐wide association study (GWAS) and whole exome sequencing (WES) studies in humans have identified various NPC susceptibility loci for HNSCC.

Trait(s)	Samples and size	Ancestry	Mapped genes	Variant and risk allele	*p*‐value	Chromosome: Position in bp	Reference
Nasopharyngeal carcinoma	1615 NPC patients and 1025 healthy	Guangdong Chinese Taiwan Chinese Malaysian Chinese	DMD	rs5927056	9.44 × 10−5	31969744	[171]
			299 kb 3′ of LOC101928201	rs6641142	3.01 × 10−4	4825493	
			REPS2	rs12860876	3.98 × 10−1	17037354	
			261 kb 5′ of CXorf51A	rs2207942	7.30 × 10−4	1.46E+08	
			FRMPD4	rs2018094	1.95 × 10−3	12272097	
			108 kb 3′ of C1GALT1C1	rs5910990	6.17 × 10−1	1.2E+08	
			43 kb 5′ of SMS	rs6528069	9.86 × 10−1	21915630	
Nasopharyngeal carcinoma			AC097658.3	rs300890‐A	9 × 10−6	4:143328617	[172]
			HLA‐A, HLA‐U	rs2860580‐?	3 × 10−31	6:29938914	
			HLA‐B, LINC02571	rs2894207‐?	2 × 10−19	6:31295974	
			HLA‐DQA1, HLA‐DRB1	rs28421666‐?	1 × 10−10	6:32624960	
			CLPTM1L	rs401681‐?	3 × 10−14	5:1321972	
			AC133065.1, CIITA	rs6498114‐?	4 × 10−9	16:10870261	
Nasopharyngeal carcinoma			HLA‐A	rs417162	2 × 10−19	6	[173]
			HLA‐A	rs2517713*	2 × 10−16	6	
			HCG9	rs9260734*	3 × 10−14	6	
			HCG9	rs5009448*	7 × 10−11	6	
			GABBR1	rs2267633	2 × 10−10	6	
			GABBR1	rs29230*	5 × 10−10	6	
Nasopharyngeal carcinoma	371 420	East Asia	ITGA9	rs189897‐A	7 × 10−8	3:37477054	[174]
Nasopharyngeal carcinoma	6034 cases/6585 controls	European, North America, South America	HLA‐A, HLA‐U	rs2860580‐?	5 × 10−67	6:29938914	[42]
			TNFRSF19	rs9510787‐G	2 × 10−9	13:23631056	
			MECOM	rs6774494‐?	1 × 10−8	3:169364845	
			CDKN2B‐AS1	rs1412829‐?	5 × 10−7	9:22043927	
			HLA‐B, LINC02571	rs2894207‐?	3 × 10−33	6:31295974	
			HLA‐DQA1, HLA‐DRB1	rs28421666‐?	2 × 10−18	6:32624960	
			TNFRSF19, LINC00352	rs1572072‐?	1 × 10−8	13:23553071	
Nasopharyngeal carcinoma	562 East Asian 2275 East Asian	East Asia	HLA‐A, HLA‐W	rs2517713‐A	4 × 10−20	6:29950322	[175]
			ZFP57, ZDHHC20P1	rs3129055‐G	7 × 10−11	6:29702484	
			SUMO2P1, MOG	rs29232‐A	9 × 10−17	6:29643654	
Nasopharyngeal carcinoma	5892	East Asian	CLPTM1L	rs31489	6 × 10−13	5:1342599	[176]
			MECOM	rs6774494	2 × 10−12	3:169364845	
			TNFRSF19	rs9510787	5 × 10−10	13:23631056	
			CDKN2B‐AS1	rs1412829	3 × 10−8	9:22043927	
			CDKN2A	rs3731239	1 × 10−6	9:21974219	
			CDKN2B‐AS1	rs4977756	7 × 10−7	9:22068653	
			CDKN2B, CDKN2B‐AS1	rs1063192	8 × 10−7	9:22003368	
			TERT ‐ MIR4457	rs2853668	4 × 10−6	5:1342599	

Several human whole‐exome/genome sequencing studies on NPC's mutational landscape have revealed multiple genetic defects related to chromatin modification and signaling pathways involving the disease's development and progression, notably pathways involving ErbB‐phosphatidylinositol‐3‐kinase (PI3K) and NF‐κB.^44^ Additionally, tumor antigen p53 (TP53) mutation is frequent in NPC, with prevalence ranging from 7.3% to 8.5%.^44^, ^45^ Nevertheless, these only account for a small portion of NPC heritability. It is imperative to conduct further association studies with larger cohorts to validate these discoveries and unveil new genetic variations associated with the risk of NPC. Fine‐mapping investigations using next‐generation sequencing technologies are needed to identify the causative variations underlying the susceptibility loci.

### 1.3 Hypopharynx SCC

Hypopharyngeal squamous cell carcinoma (HPSCC) accounts for 3%–5% of all head and neck malignancies, making it the least common HNSCC subtype. As in other types of HNSCC, the prognosis in HPSCC varies based on HPV status. The patients with HPV‐positive hypopharyngeal tumors had improved CSS compared with patients with HPV‐negative tumors. Around 60%–85% of patients with HPSCC have stage III–IV disease at diagnosis, indicating a poor prognosis despite new aggressive multidisciplinary treatments.^31^, ^46^

HPSCC possesses attributes such as its concealed location, robust infiltration, propensity for submucosal expansion, a tendency for primary lesions to grow multicentrically due to the absence of evident symptoms in the initial stages, and the deficiency of distinct indicators and reliable diagnostic measures. Most HPSCC patients are diagnosed in an advanced stage. The delayed diagnosis due to the absence of initial symptoms, the propensity for submucosal spread, the high prevalence of clinically positive nodes at presentation, and the high rates of recurrence and second primary tumors all contribute to the poor prognosis.^47^

*FGFR*1, the gene encoding fibroblast growth factor receptor 1, is emerging as a therapeutic and predictive biomarker in various cancer types, including HNSCC. Dysregulated FGFR signaling, triggered by the amplification of the *FGFR*1 gene or overexpression of FGFR1 protein, could potentially have a pivotal function in HPSCC's development and advancement, suggesting that *FGFR*1 may be a prognostic factor in disease‐free survival in HPSCC and could be a target for novel therapeutic stratiges.^47^

### 1.4 Larynx SCC

The laryngeal SCC (LSCC) is a relatively infrequent cancer,^48^ with uncertain underlying causes; smoking, alcohol consumption, low body mass index (BMI), and laryngopharyngeal reflux have all been identified as potential risk factors.^49^, ^50^ In spite of the high incidence of exposure to these factors in the overall population, only a minority develops laryngeal squamous cell carcinoma (LSCC), suggesting that genetic elements might contribute to laryngeal carcinogenesis.^51^ Furthermore, populations of varied ancestral origins exhibit genetic diversity that directly impacts susceptibility to laryngeal squamous cell carcinoma (LSCC) or influences interactions with distinct environmental factors. To date, there are only two published GWAS of LSCC. The first study was a two‐phase GWAS of non‐Hispanic whites of Chinese ancestry, and the second study identified six loci associated with laryngeal cancer risk, including four previously reported loci (2p23.1, 5p15.33, 6p21.32, and 6p21.33) and two novel loci (6p22.1 and 18q22.2: Table 1: Shete et al. 2020). These few risk loci account for a modest amount of inherited LSCC, and no more follow‐up investigations have been published. TCGA research is elucidating the genetic profile of untreated primary LSCC.^47^ Mutations in TP53, NOTCH1, CDKN2A, and PIK3CA are prevalent while HPV infection is uncommon. Meanwhile, investigations into LSCC that has recurred and/or metastasized indicate that the molecular configuration evolves during progression, encompassing a greater number of oncogenic alterations^52^; however, this association has not yet been verified in substantial cohorts of matched primary and metastatic tumors.^53^

Among the oncogenes known to participate in the formation and progression of LSCC is double mouse minute 2 homolog/mouse minute 2 homolog (mdm2). Previous research has demonstrated that overexpression of the mdm2 gene is critical for the development and progression of LSCC.^54^ Mdm2 is a proto‐oncogene (gene locus: 12q14.3 in humans) that encodes an E3 ubiquitin ligase that is localized in the nucleus and acts as a primary negative regulator in the p53‐mdm2 auto‐regulatory pathway. While aberrant mdm2 overexpression is frequently observed in breast carcinomas, data on LSCC are scarce.

### 1.5 Oral cavity SCC

Oral cancer is a prominent global malignancy, constituting 2% of all cancer instances and 44% of all cases of HNSCC, with an approximate mortality rate of nearly 50%.^16^ The most significant incidences of oral cancer were discovered in South Asian nations such as Sri Lanka, India, and Taiwan, which can be related to these countries' high rates of cigarette smoking and areca nut consumption. Together, oral and oropharyngeal cancers are estimated to account for roughly 220 000 new cases each year worldwide (5% of all malignancies).^55^

OSCC is not easy to manage and drastically impacts patients' quality of life (QOL).^56^ In contrast to other sub‐sites of HNSCC, aggressive surgical intervention coupled with adjuvant chemo‐radiation treatment remains the established benchmark for treating most oral squamous cell carcinoma (OSCC) patients.^56^ Although cancer treatments have grown rapidly, OSCC's 5‐year survival rate has consistently stayed around only 50% in recent decades.^16^ This poor prognosis is assumed to result from aggressive local invasion and metastasis, which leads to relapse. Furthermore, the absence of relevant indicators for early identification, as well as the failure of advanced lesions to react to treatment, contribute to poor OSCC outcomes.

Several GWAS have identified genes associated with OSCC, and a summary is presented in Table 1.^7^, ^57^ Previous reports in human studies show that OSCC development might be affected by multiple genetic and epigenetic changes, including deletions, point mutations, coefficient methylation, inactivation of tumor‐suppressing genes and oncogenic amplification.^58^ It may be triggered by long‐term exposure to carcinogens such as alcohol, smoking, toxic chemicals, viral infections, and inflammation.^59^ The loss of heterozygosity (LOH) on chromosomes 3p, 9p (inactivation of p16) and 17p (inactivation of p53) correlate with early‐stage oral cancer developments. In contrast, genetic changes in chromosomes 4q, 8p, 11q, and 13q are correlated with late‐stage oral carcinogenesis.^60^ At present, a limited number of GWAS (data outlined in Table 1) ave been undertaken to identify hereditary variations linked to OSCC vulnerability. These studies utilized diverse SNP arrays and employed supplementary prediction techniques. Additionally, efforts were made to pinpoint somatic mutations that might be implicated in the development of OSCC; a genome‐wide profiling study using array CGH (comparative genomic hybridization) technology was conducted by TCGA Consortium to validate CNAs (copy number alterations) on 172 OSCC samples.^61^ They discovered 26 amplifications and 3 deletions with a frequency of ≥8%. Amplifications were mapped on chromosomes 3q, 5p, 7p, 8q, 9p, 10p, and 11q, while deletions were observed in chromosomes 3p and 8p. These findings were confirmed and added to earlier evidence reported in TGCA and the International Cancer Genome Consortium (ICGC).^62^ Interestingly, this CNA pattern may offer insights into identifying which OSCC patients are at a heightened risk for lymph node metastasis. The presence of at least one of the elements of this CNA pattern was substantially related to a poor prognosis (*p* < 0.050), according to Kaplan Meier survival curve analysis.

## 2 HUMAN GENES ASSOCIATED WITH HNSCC

Genetic investigations have shown a complex pattern of with common chromosomal alterations, DNA copy number alterations, acquired mutations, and methylation of promoters,^63^ as well as considerable geographic and temporal heterogeneity.^64^ Whole‐genome sequencing investigations indicate that HPV‐negative HNSCC is linked to standard causative variables such as cigarette smoking and alcohol consumption.^65^ HPV‐positive HNSCC, on the other hand, is clinically and gnomically unique.^66^ Therapeutic breakthroughs from genetic investigations of HNSCC tend to emerge in younger patients, frequently with no previous history of smoking. Both specific and shared genomic features of HPV‐positive and HPV‐negative HNSCC have been revealed.^63^ Mutations are more common in smokers' tumors than in non‐smokers' tumors in both HPV‐positive and HPV‐negative HNSCC. Previous research has indicated that HPV‐negative tumors tend to possess a more significant number of mutations compared to HPV‐positive tumors.^63^ However, more recent research has found that the mutation rate is unaffected by HPV status.^67^ Mutations involving the tumor suppressor genes TP53 and CDNK2A are the most commonly observed somatic mutations in head and neck cancer.^68^ According to whole‐exome sequencing (WES) research, defects in genes that regulate squamous cell development, including NOTCH1, RIPK4, IRF6, and TP63, are found in over a third of malignancies.^68^

GWAS approaches have revealed common risk mutations in various complex disorders well‐powered genetic studies. To date, there are only four published GWAS of HNSCC. A study encompassing 2398 cases and 2804 controls of Chinese ancestry revealed six specific genomic loci (i.e., 5q14.3, 6p21.33, 6q16.1, 11q12.2, 12q24.21, and 16p13.2) correlated with the laryngeal cancer risk.^57^ Another study looked at the genetic susceptibility for oral cavity and pharyngeal cancer in 6034 cases and 6585 controls of European ancestry and discovered three loci (6p21.32, 10q26.13, and 11p15.4) that were linked to overall cancer risk, and four specific genomic loci (2p23.3, 5p15.33, 9p15.3, and 9q34.12) found to contribute to the risk of oral cancer. Additionally, the human leukocyte antigen (HLA) region and two other loci (6p21.32) have been associated with oropharyngeal cancer.^7^ A summary of these studies is presented in Tables 1 and 2. These few identified risk loci account for only a modest amount of heredity, and no further follow‐up investigations have been published. Similarly, the discrepancies revealed in this association analysis based on location support the heterogeneity of these tumors classified as HNSCC. Therefore, to validate these findings, it will be necessary to replicate them in separate independent populations while considering their risk factors. Furthermore, conducting additional functional studies is crucial to establish the biological framework underlying these associations.

## 3 ANIMAL MODEL FOR GENETIC HNSCC

To better understand of the processes involved in the carcinogenesis process, an optimal animal model should naturally spontaneously develop cancer in a manner similar to humans.^69^ Information obtained through the use of animal models will be critical for improvements in HNSCC therapy and detection, and several animal models have been established to investigate the pathophysiology, genetic basis, and potential therapies in HNSCC. However, no single model can recapitulate all aspects of HNSCC. Conventional carcinogenesis models, such as the hamster cheek pouch model, can help researchers better understand carcinogenesis and treatment‐preventative measures. Some previous reviews provide an overview of the standard animal models used in preclinical studies of HNSCC and discuss the value and challenges of each model.^70^, ^71^ The similarities between human and cat HNSCC are highlighted, as well as the prospect of employing spontaneous feline squamous cell carcinoma (FOSCC) as a model for HNSCC. Traditional animal models have been used in the development and evaluation of several new medicines.^71^ Different animal models of induced HNSCC have been proposed using a variety of approaches to induce HNSCC. They may be classified into three types: xenograft models, carcinogenesis caused by chemicals models, and genetically modified mouse models.^72^ The benefits and drawbacks of each type of model have already been presented.^72^ More recently, Li and colleagues and Tinhofer et al. presented a more extensive study and evaluated the advantages and disadvantages of the many mouse models established by several researchers. However, no one model developed thus far appears to be ideal for studying the pathophysiology and therapy of HNSCC.^73^, ^74^

A potent chemical carcinogen 4‐nitroquinoline‐1‐oxide (4‐NQO) has been widely utilized in mechanistic studies on the progression of HNSCC in rats and mice.^75^, ^76^ Furthermore, such a mouse model offers a platform for researching HNSCC molecular and treatment options. However, developing a mouse model of 4‐NQO‐induced malignancies takes effort and cannot easily be replicated. NQO was also observed to enhance the frequency of tongue and esophageal cancers generated by HPV16 E6 and E7 oncogenes in mice.^77^ Because none of these studies used groups of HPV16 transgenic mice without 4‐NQO treatment, the capacity of HPV16 to generate HNSCC on its own remains unknown. However, as Estêvo et al. recently demonstrated, this combination model of viral and chemical carcinogenesis has proved beneficial in partially dissecting the functions of the E6 and E7 oncoproteins in HNSCC.^78^ Although carcinogen‐induced animal models closely resemble the diverse landscape of genetic changes identified in primary human cancers,^79^ only a few of these mutations play a role in carcinogenesis by altering oncogenes or tumor suppressor genes, and most mutations are mere bystanders with no discernible impact on tumor growth. Furthermore, these investigations do not disclose if drivers are required for tumor maintenance. Preclinical model systems, such as genetically engineered mouse models (GEMMs), on the other hand, allow for a comprehensive assessment of the biological impact of particular mutations in a defined genetic context.

Genetically engineered mice are ideal models for in vivo investigations of the involvement of certain genes and genetic alterations in developing HNSCC.^74^ They provide early‐stage models that allow identification of biomarkers that are predictive and correlative for assessing various therapy methods.^13^ Investigations with transgenic and knockout mice have increased knowledge of carcinogenesis and offer more realistic models of human HNSCC in the context of an intact immune system. While several transgenic animal models have long represented the most common tumors, head and neck malignancies in genetically engineered animals are uncommon and of recent origin.^80^ While transgenic models are up‐and‐coming for HNSCC research, their limited tumor penetrance results in unpredictability of the endpoint in an experimental design.^81^ Furthermore, the tumor microenvironment within these models may diverge from that in humans, as mutations exist in all cells in knockouts and a substantial number of cells in transgenic models. A review of OSCC GEMMs was published by Ishida et al.^72^

The single‐gene knockout model of SMAD4 in head and neck epithelia (HN‐Smad4del/del) published by Bornstein and colleagues in 2009 may be the GEMM of spontaneous HNSCC most comparable to the molecular characteristics of clinical disease and has been used to analyze in depth the molecular pathways underlying HNSCC development.^82^ However, to our knowledge, it has not yet been used to develop new treatment options or to identify genetic variants involved in the pathogenesis of HNSCC.

HNSCC develops as the result of a set of genetic alterations that culminate in metastasis. In several mouse cancer models, single‐gene mutations are frequently insufficient to simulate human carcinogenesis and progression.^83^ However, most studies investigate genetic changes seen in HNSCC using a monogenetic approach. So far, only a few studies have combined *p*53 losses with *Cyclin D*1 overexpression and *P*120*CTN* downregulation with *PIK*3*CA* mutations.^84^, ^85^ These are the only HNSCC studies that have used a multiple gene alterations approach. Recently a new GEMM has been established, where mutant PIK3CA and/or *p*53 GOF alleles are conditionally expressed within the basal layer of the stratified squamous epithelium of the tongue.^86^

## 4 MODELING POPULATION DIVERSITY—THE CC MOUSE MODEL FOR CANCER RESEARCH

The impetus for developing a high genetic diversity mouse reference population resulted in the creation of the CC mouse model. This resource consists of a wide group of recombinant inbred (RI) strains developed from a genetically heterogeneous collection of eight founder strains and expressly intended for complicated trait analysis, with more power than previously described methodologies.^87^, ^88^, ^89^, ^90^

**FIGURE 2:**
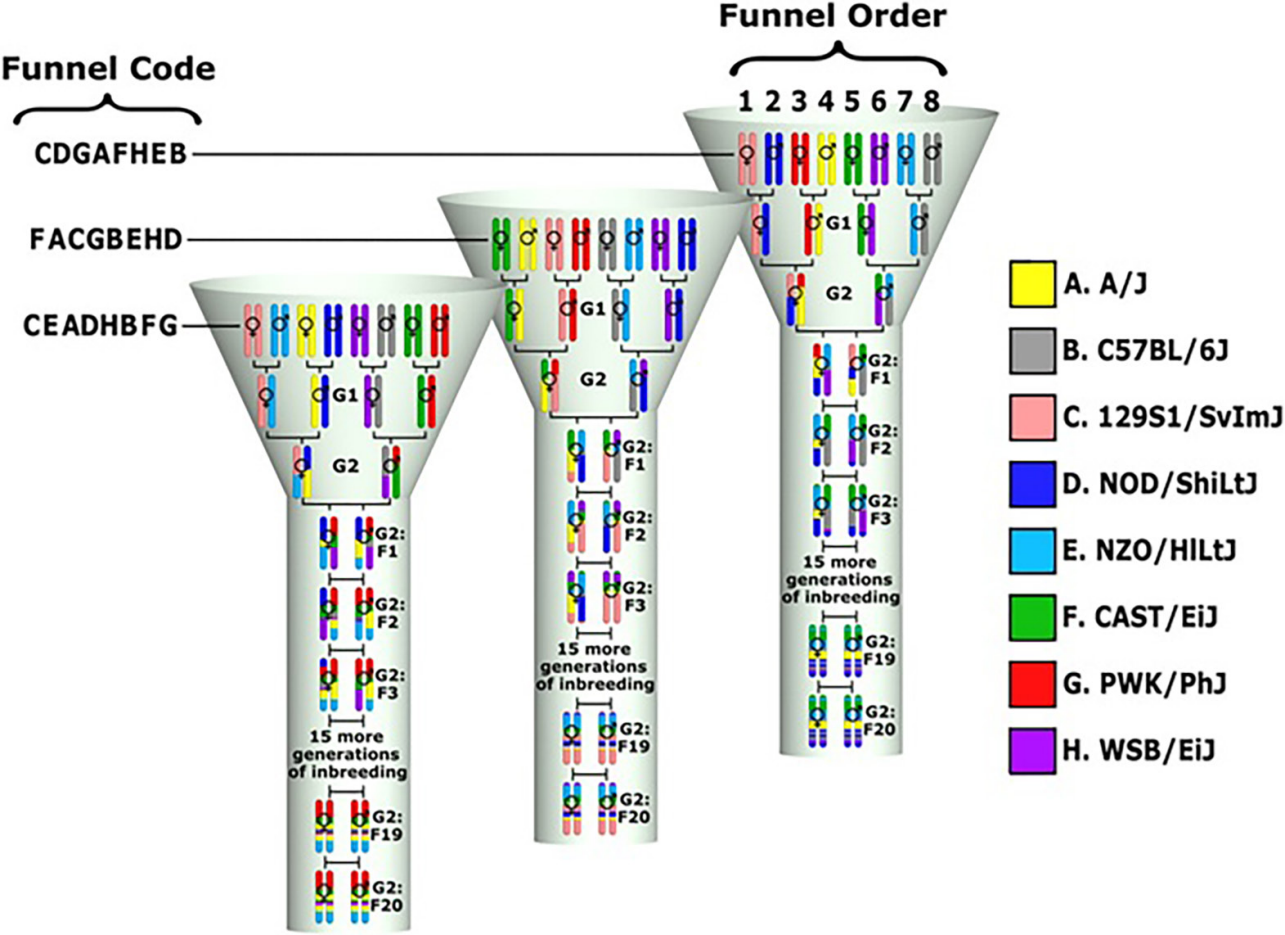
A distinctive breeding funnel scheme illustrates the creation process of the Collaborative Cross (CC) mouse model. This breeding strategy is devised to introduce genetic diversity into each inbred line. Through a single breeding funnel, one CC recombinant inbred line is produced, encompassing the genetic elements of the eight CC mouse founders. The order of the eight founder strains is randomized within each line, represented by positions 1 to 8. This order establishes the funnel code, depicted by a single‐letter code for each line. In the funnel‐breeding scheme, the genetic contributions of all eight founder strains are integrated starting from the G2 generation. A recombinant inbred line is established after 20 generations of inbreeding.^83^

Five popular laboratory strains, A/J, C57BL/6J, 129S1/SvImJ, NOD/LtJ, NZO/HiLtJ, and three wild‐derived strains, CAST/Ei, PWK/PhJ, and WSB, represent the eight founder strains (Figure 2). This evolutionary heterogeneity ensures a final population of CC mice with a significant genetic diversity that other mouse models lack.^86^, ^91^, ^92^

**FIGURE 3:**
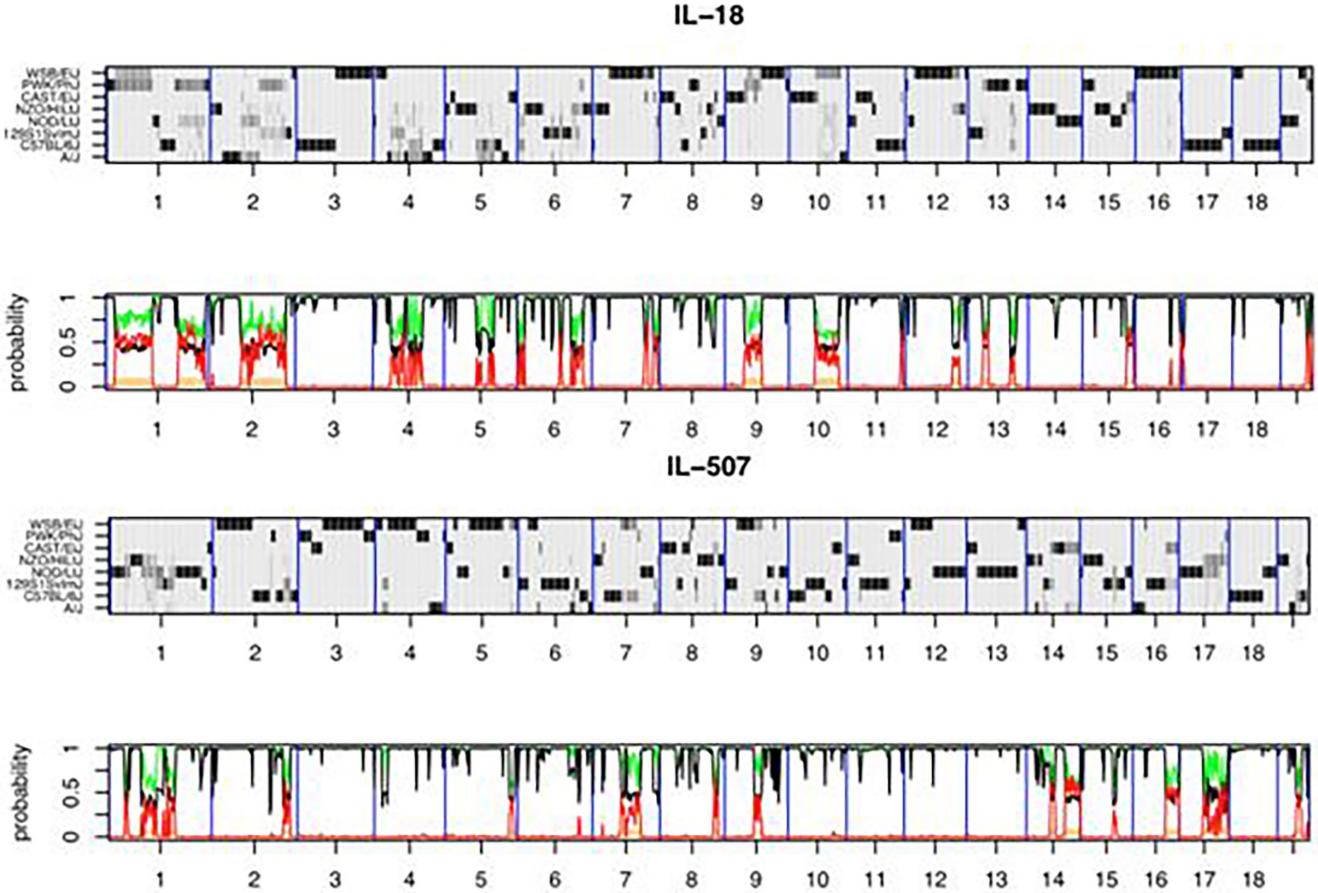
The genomes of CC lines IL‐18 and IL‐507 were reconstructed from the hidden Markov model (HMM) using HAPPY. The *x*‐axis illustrates the 19 autosomes. Each reconstruction is presented in two panels: the top panel's *y*‐axis displays the 8 CC founders, and the probability of descent from a founder at a specific locus is depicted by varying shades of gray, with white representing 0 probability and black representing a probability of 1. Dark horizontal bands indicate regions where a single haplotype is dominant, while loci with residual heterozygosity or indistinguishable founder haplotypes are shown in paler gray. The lower panel displays local heterozygosity in red, the posterior probability of the most probable founder in black, and the sum of the most probable pair of founders in green.^85^

Figure 2 shows how the genomes of all CC founder strains are incorporated into a single CC line using a well‐planned breeding methodology. In the first step, three generations of outbred matings create litters with all potential genomic alterations. This phase aims to collect recombinant events, reorganize founder genomes, and represent all eight founder haplotypes in newly created CC lines. The next stage is inbreeding, brother–sister mating, which continues for nearly 20 generations to achieve around 99% homozygosity with nearly equal contributions from the eight founder strains.^93^, ^94^ Litters from 20 and more inbreeding generations will be syngeneic (have the same genetic composition), which will benefit future research. By rearranging the founder strains that engage in the outbred mating phase, an entirely different genetic mosaic can be generated in a new CC line. As a result, each CC line's genetic component is distinct. A genetic reference population (GRP) was created using this breeding system (Figure 2), which is defined as sets of syngeneic individuals with fixed and known genomes (Figure 3). The new genetic reference population (GRP) now reflects a large proportion of the genetic diversity seen in wild populations.^95^, ^96^, ^97^, ^98^

The CC mice population is advantageous for gene mapping because of the large number of genetic variations segregating in the population (over 36 million SNPs) and the high degree of recombination events compared to other mouse populations (4.4 million SSNPs segregate between the founders). Recent papers have shown that the quantitative trait locus (QTL) mapped in CC mice tends to map contrasting alleles between wild‐derived lines and laboratory lines.^99^, ^100^ QTL analysis in the CC population has revealed that the mapping resolution is <1 Mb. The CC population's distinct properties allow for investigation of the complex genetic etiology of human illness, as well as interactions between genetic and environmental variables.

Examples include research into genetic resistance to viral pathogenesis and the role of the immune system in cancer onset and progression.^23^, ^101^ Certainly, the CC strains offer a distinctive avenue for researching cancer prevention. They enable the fusion of population genetics with the impact of tumor drivers and/or environmental influences, as demonstrated in the cases of melanoma, prostate cancer, and intestinal cancer.^23^, ^101^, ^102^ For instance, crossing the transgenic adenocarcinoma mouse prostate model of deadly prostate cancer into the eight founder strains of the CC demonstrated tumor and metastatic phenotypic modification in particular strains. Although it did not address cancer prevention, this example highlights the potential of modeling population diversity and examining its implications for cancer in vivo.^103^

### 4.1 Prospects involving the creation of a novel model to map genes associated with HNSCC using the CC model

**FIGURE 4:**
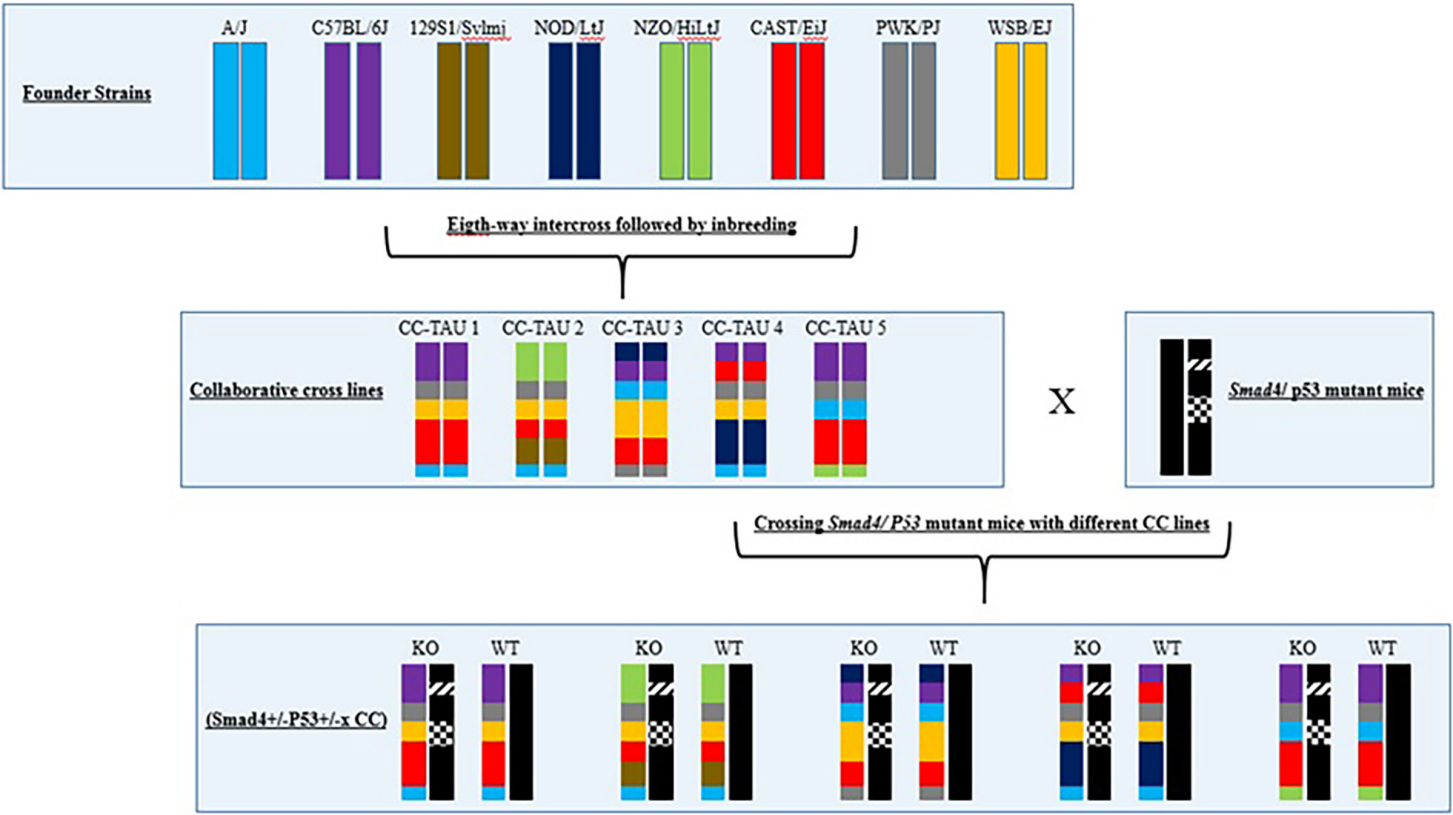
A breeding scheme involving generation of F1 mice (Smad4ko × CC). The various colors represent the genotypes of the eight founder strains' respective chromosomes. The CC lines produced (designated as CC1, CC2, CC3, CC4, etc.) are then mated with Smad4 and P53 mutant inbred mice, yielding an F1 population (Smad4+/−P53+/− × CC) along with a control F1 population (Smad4+/+P53+/+ × CC). In the F1 (Smad4/P53 × CC) mice, one chromosome originates from the Smad4 mutant inbred mice, while the second chromosome comes from the individual CC line. Any variations in phenotype and genotype among the F1 crosses from distinct CC lines are solely attributed to the CC genotypes. CC, Collaborative Cross; KO, knockout; WT, wild‐type.

To acquire a better knowledge of the genetic components involved in tumor initiation and progression, a new HNSCC model needs to be developed that considers the human population's diversity. Crossing a transgenic HNSCC mouse model with a CC mouse model may shed new light on the genetic underpinnings of the HNSCC disease. As previously stated, the most similar GEMM to spontaneous HNSCC is the single‐gene deletion model of *Smad4* in head and neck epithelia. Thus, mating Smad4 mutant mice with various CC lines having high genetic diversity theoretically results in an F1 (Smad4‐ × CC) with dramatically varying susceptibility to spontaneous HNSCC formation (Figure 4).

Briefly, the Smad4 gene was discovered to be a tumor suppressor in pancreatic carcinoma and was later recognized as a critical modulator of TGF signaling.^104^ Smad4 participates in TGF/BMP signaling by building complexes with receptor‐activated Smads, such as Smad2 and 3 or Smad1 and 5. Smad complexes subsequently translocate to the nucleus, where they influence gene expression of Smad targets implicated in several cancer‐related processes like proliferation, apoptosis, and inflammation.^105^ Smad4 somatic silencing is frequently encountered in a variety of tumor types.^105^ Moreover, Smad4 deletion in mouse tissues, together with other genetic changes that trigger tumor growth, resulted in cancer lesions in colon,^106^, ^107^ pancreas,^108^ stomach,^109^ and liver.^110^ These outcomes are often linked with worse histological differentiation and a worse clinical outcome.^111^, ^112^ Thus, Smad4 deficiency plays a crucial role in cancer development. Furthermore, Smad4 deletion in mice led to the genesis of spontaneous stomach cancer,^113^ skin,^114^ and mammary gland^115^; Smad4 deficiency appears to have both initiating and promoting effects on cancer in these tissues. Mice with Smad4 deletion in T cells developed gastrointestinal inflammation and lower intestine cancers resembling juvenile polyposis (JP), as well as oral SCCs.^116^

Considering the function of *Smad*4 in HNSCC development, the Smad4 region on chromosome 18q is commonly deleted at the genetic level in HNSCC cases.^117^ Reported loss of heterozygosity (LOH) at the *Smad*4 locus has been documented in almost half of human OSCC patients.^9^, ^10^ Additionally, SMAD4 is associated with invasion depth, pathologic stage, localized metastases, and lower survival.^118^, ^119^

In human HNSCC, Smad4 expression was reduced in 86% of tumors and 67% of surrounding nonmalignant mucosa, while a Smad4‐deficient mouse model demonstrated enhanced HNSCC susceptibility.^10^, ^82^ In ten Smad4+/ mice, Redman et al.^120^ observed the development of hyperplasia, neoplasia, and other abnormalities. Further, it has been demonstrated that heterozygous Smad4 mutations, combined with other genetic variants, lead to widespread hyperplasia and neoplastic development in various organs, including the head and neck region.^82^ These results reveal that the Smad4 gene enhances the initiation of HNSCC and accelerates the progression of oncogene‐driven HNSCC.

Producing new NHSCC mouse models based on this information, with the benefits of diversity and tumor heterogeneity found in the CC model, will enhance understanding of how diverse genetic changes cause HNSCC initiation and progression and what subtype of cancer will develop.

Considering that human carcinogenesis occurs in the context of numerous gene mutations, research into multigene co‐expression should be emphasized in cancer research. For instance, overexpression of *EGFR* and downregulation of *P120CTN* contribute to the enhanced invasion of esophageal squamous cell carcinoma.^83^ Furthermore, while overexpression of Cyclin D1 alone does not induce invasion in HNSCC, co‐expression of *p5*3 results in a more invasive phenotype.^84^ These findings encourage us to investigate the potential involvement of *Smad4* via similar mechanisms in HNSCC development.

Somatic mutations targeting the tumor suppressor gene *TP*53 are the most frequently reported genetic variations in head and neck cancer. Mutations in the *TP*53 gene, which encodes p53, are widespread in HPV‐negative cancers. Disruptive alterations, particularly to TP53, have been related to poor prognosis and therapeutic resistance. Thus in patients with HNSCC, p53 mutations are related to increased survival and higher insensitivity to radiation and chemotherapy.^121^ We therefore suggest investigation of the tumorigenic implications of a p53 mutation in addition to Smad4 knockout. Because p53 gene mutations are prevalent in HNSCC with an LOH of 17p and TP53 point mutations are detected in 40%–50% of premalignant lesions,^122^ we expect that an extra p53 mutation will increase vulnerability and collaboratively induce carcinogenesis, as has been observed in other tissues like the mammary gland.^123^

**FIGURE 5:**
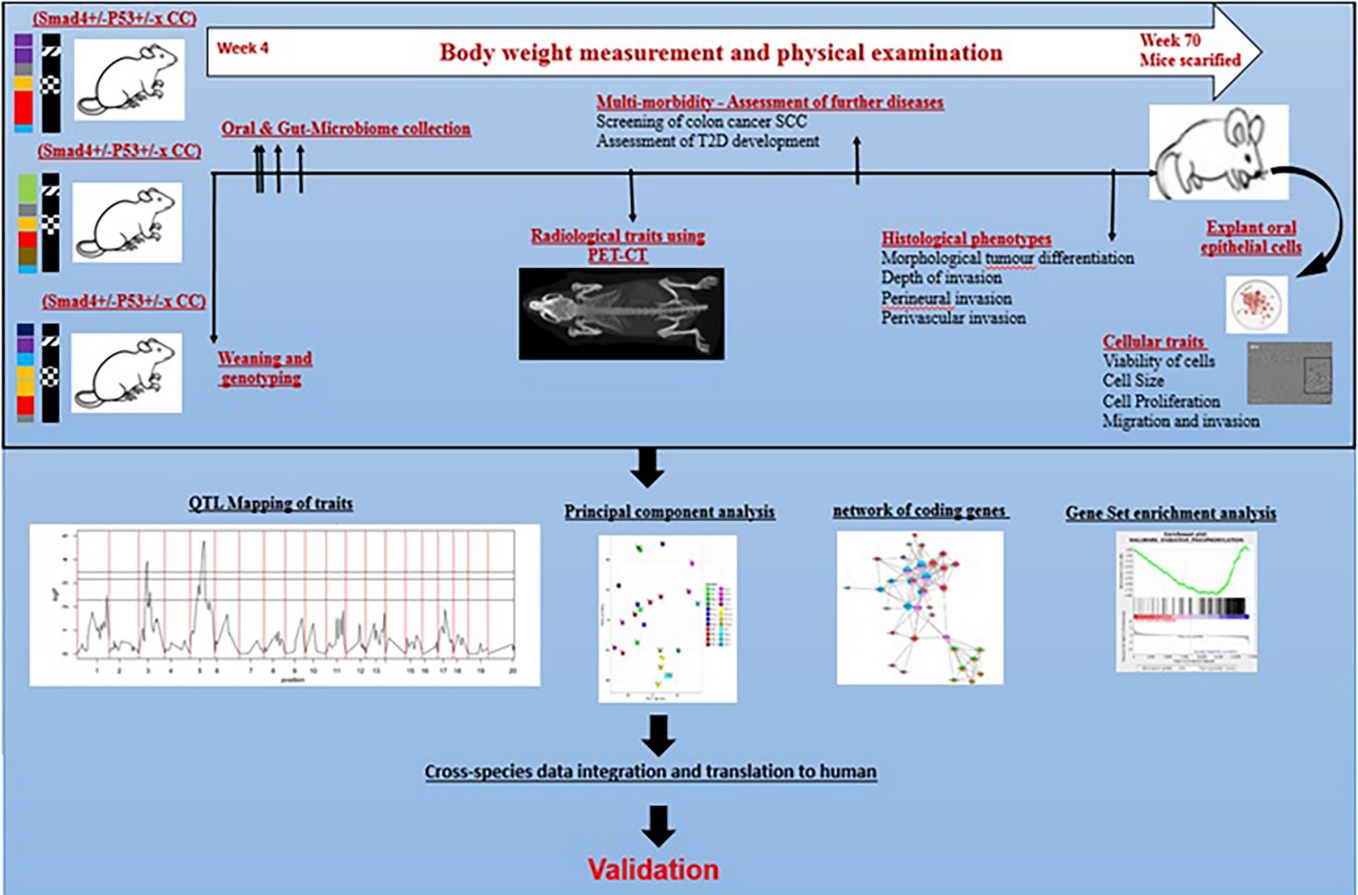
Workflow for producing a systems genetics data set from the high diversity F1 (Smad4/P53 × CC) population, which may differ greatly in susceptibility to OSCC development. Mice are evaluated for several OSCC‐related clinical and histological characteristics. Primary cell cultures are produced from the F1 (Smad4+/−P53+/− × CC) population and evaluated for several cellular characteristics. Data from cellular, molecular, and clinical features are then pooled to examine various HNSC‐phenotype connections. With QTL mapping, the regulatory genomic areas implicated in phenotypic variation in both in vitro and in vivo monitored variables may be discovered by combining SNP genotype data from each CC line. Combining the data with later candidate‐gene association studies in people provides the potential to find susceptibility genes linked to OSCC development in humans.

The strategy for generating system genetics data and phenotypic sets from a high diversity F1 (Smad4/P53− × CC) population, which may differ and increase significantly in their susceptibility to HNSCC development, is shown in Figure 5. Mice are evaluated for various clinical and histological characteristics associated with HNSCC. Subsequently, the effect of *Smad*4 and P53 knockout on tumorigenicity in a large group of cells from different genetic backgrounds using primary cell cultures obtained from the F1 generation (*Smad*4/P53 CC) is investigated.

Data from cellular–molecular populations are tested for various cellular properties using the IncuCyte Live‐Cell Analysis System. This system uses unique software and clinical features to automatically take and evaluate photos of live cells based on algorithms for each cell line. These are pooled to investigate various HNSCC‐phenotype associations. By incorporating SNP genotype data from each CC line, QTL analysis can identify regulatory genomic regions associated with variations in both in vitro and in vivo monitored characteristics.^124^, ^125^, ^126^, ^127^

Integrating these data with later candidate‐gene association studies in people will provide an opportunity to identify susceptibility genes related to HNSCC development in humans. For such studies, the capacity to undertake parallel in vitro/in vivo screening, as well as advancements in high‐throughput assessment technologies and computational approaches, is essential. This might lead to an improved understanding of how the mix of genetic changes affects the onset and severity of HNSCC. Further evidence showing how gene‐phenotype interaction and/or a gene–environment complex applies to human systems will help guide the development of treatments for HNSCC or potential drug targets. Systems genetics is anticipated to assist in better understanding of both biological mechanisms and illness intensity because of the processes associated with the genetic loci (QTL and genes) revealed by GWAS.

In order to comprehend the pathophysiology, transmission, and host immune response to infection, animal models are essential.^128^ Furthermore, the development and testing of medicinal drugs and vaccines depend heavily on animal models.^129^ Before choosing an animal model to study an infectious agent, the scientific team must first decide if there is enough ex vivo and in vitro data available to support conducting research into an animal model, if ethical issues are taken care of, and if the data obtained from animal work will contribute meaningfully to the body of scientific knowledge.^130^, ^131^, ^132^ In addition, in selecting an animal model, the animal's innate vulnerability to the virus under investigation should be taken into account. Animal models with natural disease transmission pathways, pathogenesis, and clinical illness progression that closely resemble the original host (humans, for example) are frequently preferred, depending on the goals of the research. This kind of model has been described as a ‘natural model’. The influenza virus in ferrets,^133^ the *Mycobacterium tuberculosis* ^134^ in macaques, the rabies virus in dogs^135^ and fish,^136^, ^137^ and *Brucella abortus* in goats^138^ are among the examples. In order to create the disease state, these models require artificial manipulation of the host and the infectious agent. The models make it easier to research virulence factors, pathways of transmission, and the host immunological response, which includes gene expression regulation.^139^, ^140^ Additionally, these models may allow for the use of a lower dose of infectious organisms, improving researcher safety compared to other model types. For research on infectious diseases, mice are frequently used as the model species of choice. In addition to their tiny size, accessibility, ease of handling, and relative affordability, they are available as both inbred strains and outbred stocks. Mice are easily genetically engineered, can contract numerous human infections, and have a well‐defined immune system that is comparable to that of humans.

There are also drawbacks. It cannot be assumed that research on animals will yield comparable results in humans. Thus, when applying the results of well‐known animal studies to the treatment of human illness, patients and doctors should exercise caution.^141^ Results from even high‐caliber animal studies will not replicate well in clinical research on humans. Use of animal experiments can impede or delay discovery—when treatments and medications fail to work in animal trials, they may never be developed for human use. Since human trials of medications that fail in animals are rare, it is impossible to determine how frequently this happens, but there have been a few noteworthy instances. Numerous variables that may complicate human investigations are automatically controlled in the design of animal studies. Studies on animal phenotypes are generally limited in scope and do not evaluate the impact of naturally existing variation on phenotypes; hence, the phenotypes are not exactly the same as the human disease.^142^

## 5 EPIGENETIC REGULATION AND HNSCC

Epigenetics is the study of genetic data transmission based on gene activity without changing the genetic code.^143^ Epigenetic mechanisms of regulation are required for early growth and tissue‐specific gene expression. Alterations and epigenetic abnormalities are considered early hallmarks of cancer. Epigenetic changes comprise changes in DNA methylation, histone modifications, nucleosome positioning, and miRNA expression.^144^

## 6 DNA METHYLATION

The most common epigenetic change identified in humans is DNA methylation, a heritable and enzyme‐mediated chemical modification of DNA structure with no alteration of the nucleotide sequence.^145^ The methylation process catalyzes DNA methylation and is essential for regulating gene expression, that is genes silenced on the X chromosome.^145^ The most thoroughly investigated epigenetic change has been DNA methylation, whereby a methyl group (CH3) is covalently bonded to the 5‐carbon on cytosine and generally connected to guanine through a phosphodiesterase link, forming CpG islands, primarily located in promoter regions or the first exon of a gene.^145^ In the history of cancer epigenetics, DNA methylation is an essential process that controls the expression of genes and the stability of the genome. DNA methyltransferase enzymes (DNMT) mediate DNA methylation alterations. Several DNA methyltransferase enzymes have been identified by knockout studies, but each poses unique functional activity. DNA methylation can lead to decreased gene expression by blocking transcription machinery and stimulating histone modifications that lead to the formation of tightly packed heterochromatin.^146^, ^147^

Alterations in methylation patterns play a role in the development of neoplasia and the expansion of tumors.^148^, ^149^ Promoter hypermethylation of tumor suppressor genes in tumor tissues is a joint event in developing many types of cancer, including HNSCC.^150^, ^151^, ^152^ This leads to a loss of function that promotes cell proliferation.^134^ Proto‐oncogenes are mostly hypomethylated in several cancers.^153^

## 7 DNA HYPOMETHYLATION

Systemic genetic hypomethylation in tumor tissue is a typical characteristic of tumors ranging from solid tumors to blood cancers.^154^ Global hypomethylation in repetitive sequences, intron CpG islands, and transposons affect genome stability and activate oncogenes.^149^, ^155^ Although the earliest epigenetic modification was reported in cancer, it was not extensively investigated until many studies had reported the significant increase in global and cancer‐irrelevant gene hypomethylation in various cancers.^156^, ^157^

Multiple studies have reported and investigated hypomethylation patterns in HNSCC,^158^, ^159^ including methylation patterns of repetitive sequences, such as long interspersed nucleotide element‐1 (LINE‐1) and short interspersed nucleotide element‐1 (SINE‐1). The molecular and cellular associations of hypomethylation reported in various studies are presented in Table 3.

**TABLE 3:** Hypomethylated repetitive sequences in HNSCC.

Cancer	IRS	Molecular or cellular associations	Significance	Reference
HNSCC	LINE‐1	Advanced clinical stage	*p* = 0.003	[177]
HNSCC	LINE‐1	Reduced survival rate	*p* = 0.04	[178]
HNSCC	LINE‐1, Alu and Sat‐α	Dismal prognosis		[179]
OSCC	Alu	Advanced stages have more methylation		[150]
OSCC	LINE‐1			[180]
OPSCC	LINE‐1	3.5 fold relapse risk		[181]
TSCC	LINE‐1	Linked to poor survival rate in females		[181]

The global methylation pattern was assessed using the LRE1 method as a whole blood biomarker for HNSCC in 278 subjects. The results revealed that the median methylation level in HNSCC cases was significantly higher than the median level in controls (*p* < 003) and suggested an increased risk of HNSCC when LER1 was hypomethylated after controlling for other risk factors and confounders.^144^

Multiple studies have reported promotor hypomethylation in various genes, that is *WSIP*1 overexpressed in OSCC patients with lymph node metastasis.^160^ The *CSPG4* promoter region was reported to be hypomethylated in HNSCC, which was linked to worsening clinical phenotype.^161^ Promoter methylation pattern alteration of PD‐L1, PD‐L2, TR, EX2, and MSH4 was also mentioned in previous reports of HNSCC,^52^, ^162^ while promoters of IL6, PTPN22, RUNX1, CD28, CD22, TLR,1 and TNFa were shown to be hypomethylated in OSCC.^163^

## 8 DNA HYPERMETHYLATION

Transcriptionally active genes in normal cells are usually hypomethylated. Hypermethylation of the CGIs of the promoter regions of some genes is considered a characteristic of cancer tissue; it mostly occurs in tumor suppressor genes, and may lead to silencing of these genes and induce malignant transformation.^147^ The multiple reports of hypermethylated promoters were detected by multiple studies in various types of HNSCC, some of them are presented in Table 4.

**TABLE 4:** Hypermethylated genes in HNSCC.

Cancer	Gene	Molecular or cellular associations	Reference
OSCC, HNSCC	p16	Associated with male gender as well as with LN metastasis	[182, 183]
OSCC, NPSCC	PTEN	Well‐differentiated tumors	[179, 184]
HNSCC	DAPK	Lymph node involvement and linked to advanced stage of HNSCC	[185]
HNSCC	MGMT	Advanced stages have more methylation	[186]
HNSCC	RASSF1		[187]
HNSCC, OSCC	E‐cadherin		[188, 189]

## 9 NON‐CODING RNAs

Nonprotein‐coding RNAs are classified into multiple categories according to their molecular size: small noncoding RNAs, including miRNAs and siRNA, and long noncoding RNAs. Non‐coding RNAs play a critical role in epigenetic regulation in normal cells, facilitating normal development and differentiation. Alterations of non‐coding RNAs lead to the development of multiple diseases, including cancer.^164^

## 10 miRNA

miRNA is a short non‐coding single‐stranded RNA, about 25 nucleotides in length. The sequence of miRNA is usually complementary to the 3′‐untranslated region of messenger RNAs; when it binds to the mRNA, it causes its degradation or stops the translation machinery; about 30% of the human genes are regulated by miRNAs.^164^

miRNAs participate in the regulation of many cellular processes like proliferation, differentiation, development, and apoptosis. Thus miRNA alterations have been intensively studied as an epigenetic biomarker of cancer. These miRNAs may be classified as oncogenes or suppressor genes for cancer‐related expression and their target action gene.^165^ Some miRNAs altered in HNSCC are presented in Table 5.

**TABLE 5:** Collection of microRNA (miRNA) expression profiles of HNSCC.

miRId	Family/cluster	Profile
hsa‐let‐7d		Down
hsa‐let‐7d		Up
hsa‐miR‐1		Down
hsa‐miR‐1		Down
hsa‐miR‐1		Down
hsa‐miR‐106b	mir‐106b‐25	Up
hsa‐miR‐107		Down
hsa‐miR‐107		Down
hsa‐miR‐125a‐5p		Up
hsa‐miR‐128		Down
hsa‐miR‐133a		Down
hsa‐miR‐133a		Down
hsa‐miR‐134		Up
hsa‐miR‐138		Down
hsa‐miR‐138		Down
hsa‐miR‐138		Down
hsa‐miR‐141		Down
hsa‐miR‐149		Down
hsa‐miR‐150‐3p		Down
hsa‐miR‐150‐5p		Down
hsa‐miR‐16		Up
hsa‐miR‐182		Up
hsa‐miR‐184		Up
hsa‐miR‐196a		Up
hsa‐miR‐200c		Down
hsa‐miR‐203		Down
hsa‐miR‐204		Down
hsa‐miR‐204		Down
hsa‐miR‐205		Down
hsa‐miR‐205		Up
hsa‐miR‐206		Down
hsa‐miR‐206		Down
hsa‐miR‐21		Up
hsa‐miR‐21		Up
hsa‐miR‐21		Up
hsa‐miR‐21		Up
hsa‐miR‐218		Down
hsa‐miR‐223‐3p		Up
hsa‐miR‐25	mir‐106b‐25	Up
hsa‐miR‐27a*		Down
hsa‐miR‐29a	mir‐29	Down
hsa‐miR‐29c	mir‐29	Down
hsa‐miR‐300		Down
hsa‐miR‐30a‐5p		Up
hsa‐miR‐31		Up
hsa‐miR‐34a		Down
hsa‐miR‐363		Down
hsa‐miR‐372		Up
hsa‐miR‐375		Down
hsa‐miR‐375		Down
hsa‐miR‐375		Down
hsa‐miR‐422a		Down
hsa‐miR‐451		Down
hsa‐miR‐675		Up
hsa‐miR‐874		Down
hsa‐miR‐876‐5p		Down
hsa‐miR‐9		Down
hsa‐miR‐93	mir‐106b‐25	Up
hsa‐miR‐93		Up
hsa‐miR‐96‐5p		Up

## 11 lncRNA

lncRNAs are approximately 200 nucleotides in length, and do not code for any protein product. lncRNAs positively or negatively alter epigenetic regulation at multiple gene expression levels and are considered pivotal regulators of multiple biological processes such as apoptosis, metabolism, proliferation, and cell cycle.^166^ Since lncRNAs are contributors to multiple cellular processes that are altered in cancers, the effect of lncRNAs has been studied in cancer progression and tumor formation and as a biomarker in various cancers, especially HNSCC.^167^ An example of altered lncRNA in HNSCC is the up‐regulation of HOXA11‐AS. This upregulation was correlated with poor prognosis in LSCC, and it was linked to lymph node involvement and metastasis in OSCC.^168^ Down‐regulation of HOXA11‐AS in cell lines resulted in decreasing invasion and cell migration, suggesting an oncogenic role of HOXA11‐AS.^169^ Some lncRNA may also function as tumor suppressor lncRNA. LINC01133 was reported to be downregulated in OSCC, while regular expression was linked to longer survival rates in OSCC.^170^

### 11.1 Histone modifications

The main unit of the chromatin, the nucleosome, comprises four histone proteins (two units of H2A, H2B, H3, and H4) that form a histone octamer with 150 nucleotide pairs of DNA coiled around it. Histones are structural proteins consisting of a globular C domain and N terminal tail, where the N terminal domain undergoes numerous covalent changes, including acetylation, methylation, phosphorylation, and ubiquitination. Histone post‐translational modifications modulate histone structure, which alters the DNA histone interactions altering chromatin structure and thus the transcriptional activity. Histone post‐translational modifications play a central role in gene expression control. Alterations of histone modifications are linked with various cancers, including HNSCC.^166^

### 11.2 Histone acetylation

The most significant alteration of histones involved in gene expression, chromatin architecture, and DNA damage repair is lysine acetylation. The acetylation process results from a balance between histone acetyl transferases and histone deacetylases. The acetylation status of the histone lysine influences nucleosome confirmation; acetylation results in a more relaxed nucleosome structure, facilitating gene transcription and vice versa.^166^ Altered acetylation status can result in enhanced transcription of numerous genes (i.e. oncogenes), leading to malignant transformation.^171^ In HNSCC cells, chromatin hypoacetylation occurs. This is indicated by a decrease in the amount of histone H3 acetylated on lysine 9 (H3K9) compared to standard oral keratinocytes.^172^ Histone H4 acetylated on lysine 16 is associated with early clinical stages of cancer in HNSCC patients, whereas histone H3 acetylated on lysine 9 is associated with early clinical stages as well as rising degrees of differentiation and the lack of lymph nodes.^171^ OSCC patients exhibit hypoacetylated H3K9ac, and this condensation of chromatin is related to a reduced survival rate, whereas H3K4as was linked with nodal invasion and decreased survival.^173^

### 11.3 Histone methylation

The methylation of the amino acid residues (lysine, histidine, and arginine) is effected by histone methylases, while histone demethylases counteract this modification. Methylation of histones contributes to alterations in chromatin structure and gene regulation, while leaving the histones' charge unchanged.^166^ The impact of methylation on epigenetics varies depending on the specific site of the methyl group addition. Methylation of H3K4, H3K79, and H3K36, for instance, leads to a more open and relaxed chromatin structure, which is a marker of transcriptionally active loci. In contrast, the methylation of H3K9 and di and trimethylation of *H3k*27 causes the condensation of chromatin (silenced).^166^ A high level of H3K27 methylation is linked to tumor progression and lower survival rates in OSCC. Furthermore, dimethyl H2K4 instead of trimethylated H3K4 has been reported in OSCC.^173^, ^174^

Polycomb proteins serve an essential function in chromatin remodeling and epigenetic control. There are multiple lines of evidence suggesting a correlation between HNSCC and polycomb proteins.^166^ EZH2 belongs to the polycomb group responsible for the methylation of H3K27 and, thus, gene silencing. EZH2 expression is higher in OSCC cell lines than in dysplasia or normal mucosa cells. Furthermore, greater EZH2 mRNA expression has been found in HNSCC patients and is associated with stages of cancer and relapse, implying a role for EZH2 in HNSCC progression.^175^

### 11.4 Histone phosphorylation

The incorporation of a phosphate group changes the charge on the histone; it becomes less positive. This leads to nucleosome structural changes and nucleosome remodeling. Phosphorylation occurs mainly on the amino acids threonine, serine, and tyrosine in the N‐terminal histone tails and is controlled by kinases and phosphatases.^166^ Indications of altered phosphorylation patterns were reported in HNSCC in multiple studies. The ARK2, which is a threonine protein kinase of H3S10, was found to be over‐expressed in HNSCC patients and was associated with histological differentiation, cell growth, and metastasis in carcinoma of the oral cavity. Its overexpression was also linked to poor survival, and cytoplasmic overexpression was linked to T status and tumor staging.^171^, ^172^

### 11.5 Histone sumoylation

Sumoylation is analogous to ubiquitination because sumoylation enzymes add molecules of the tiny ubiquitin‐like modifier (SUMO). This is also reversed by sumo‐specific proteases (SENPs). Histone sumoylation leads to its lysis and, of course, alteration of the chromatin structure.^166^ Some reports suggest a link between HNSCC and sumoylation. Other studies reported overexpression of sumoylation enzymes in HNSCC, a linked variation in the expression of these enzymes with the clinical phenotype of the cancer.^172^

## 12 PROTEOMICS OF HNSCC

Proteomics is the study of proteomes on a vast scale. A proteome is a collection of proteins made by an organism, system, or biological milieu. Measuring variations between two or more physiological states of a biological system is one of the most essential but also one of the most complex technical challenges in proteomics. There have been efforts to identify secreted biomarkers from serum or plasma to detect various cancer types. However, such body fluids have produced limited results due to their enormous intricacy and substantial dynamic range. Many biological processes related to cancer metastasis and progression are promoted by secreted proteins from cancer cells or tissues, making secretome a potential biomarker reservoir. There is no specific biomarker that can be utilized to identify and monitor disease development in HNSCC patients. To enhance the clinical outcome of HNSCC patients, detection markers are required. These should be non‐invasive and hence not require a tissue sample. The use of mass spectrometry for global analyses of protein expression and secretion has grown in popularity in recent years. Identifying changed protein expression in HNSCC tissues may provide an impartial method of identifying critical biological processes, leading to a better knowledge of disease progression.^176^

There have been multiple attempts to identify biomarkers for HNSCC from cultured cell lines. Cell line models are well suited to such studies, as they are an easy‐to‐manipulate system that allows modulation and quantification of differences in secreted proteins between cancer cells in an in vitro system under controlled conditions. In a study of the secretomes of OSCC cell lines, 225 differentially secreted proteins were identified; among the upregulated proteins were OLFM4, IGFBP3, HGFAC, and ITIH2.^177^ In another study to detect candidate biomarkers for HNSCC, in which proteins were detected in cell lines as well as in tumor tissues, a bioinformatics tool was used to detect the presence of signal sequences to make sure that the protein is part of the secretome. In this study, 132 proteins of interest were found within the OSCC tissue proteome (*p* = 0.01; more than 2 are upregulated). These proteins are likely to be secreted (detected in all 9 cancer cell line secretomes with an average abundance of over 5 counts), and the findings suggest their potential application as non‐invasive biomarkers. Out of 132 candidates, 106 have been recognized as potential biofluid indicators for OSCC based on salivary proteome data from normal mucosa versus OSCC datasets and the normal salivary proteome database, and 25 top candidates were identified in all three investigations, with THBS2, LGALS3BP, and DNAJB11 being potentially helpful salivary markers for the identification of HNSCC.^178^

A mass spectrometry (MS)‐based proteomics study of urine samples from HNSCC patients before and after therapy identified proteins differently released in tumor patients' urine. This strategy allowed the discovery of numerous proteins associated with inflammation and cancer that might be used as tumor biomarkers. Emanuele Ferrari et al. investigated urine proteome alterations between and following therapy. Fourteen proteins were discovered to be more prevalent in cancer patients' urine, while six proteins were found to be greater in controls.^179^

## 13 CONCLUSION

Several alleles linked with HNSCC have been proposed based on human association studies. The combined effects of these alleles are insufficient to explain most heritable disease risk, which shows that the genetic influence on HNSCC susceptibility cannot be elucidated exclusively through methods for determining the main effects of individual alleles in human populations. Epigenetic mechanisms also play an essential role in HNSCC development, especially alterations to DNA methylation. Histone modifications may participate in cancer progression by altering chromatin structure. There is an urgent need to introduce a novel model. As suggested in this report, this should be as close to human HNSCC as possible in order to advance knowledge of this disease and eventually lead to a better prognosis through improved treatment.

## AUTHOR CONTRIBUTIONS

Conceptualization: Fuad A. Iraqi, Aysar Nashef, and Osayd Zohud; Methodology: Osayd Zohud, Iqbal M. Lone, and Fuad A. Iraqi; Validation: Fuad A. Iraqi; Investigation: Iqbal M. Lone, and Osayd Zohud; Resources: Fuad A. Iraqi; Data Curation: Osayd Zohud, Iqbal M. Lone, and Fuad A. Iraqi; Writing—Original Draft Preparation: Osayd Zohud, and Iqbal M. Lone; Writing—Review and Editing: Osayd Zohud, Iqbal M. Lone, and Fuad A. Iraqi; Supervision: Fuad A. Iraqi; Project Administration: Fuad A. Iraqi; Funding Acquisition: Fuad A. Iraqi and Aysar Nashef; All authors have read and agreed to the published version of the manuscript.

## FUNDING INFORMATION

This study was supported by a core fund from Tel Aviv University and the Department of Oral and Maxillofacial Surgery, Baruch Padeh Medical Center, Poriya, Israel.

## CONFLICT OF INTEREST STATEMENT

The authors declare no conflicts of interest.Fuad A. Iraqi is an Editorial Board member of AMEM and a co‐author of this article. To minimize bias, he was excluded from all editorial decision‐making related to the acceptance of this article for publication.

## INFORMED CONSENT STATEMENT

Informed consent was obtained from all subjects involved in the study.

## Data Availability

Data are contained within the article.
